# Radiology Reporting System Data Exchange With the Electronic Health Record System: A Case Study in Iran

**DOI:** 10.5539/gjhs.v7n5p208

**Published:** 2015-03-16

**Authors:** Maryam Ahmadi, Marjan Ghazisaeidi, Azadeh Bashiri

**Affiliations:** 1 Health Information Management Department, School of Health Management and information Sciences, Iran University of Medical Sciences, Tehran, Iran; 2 Health Information Management Department, School of Allied-Medical Sciences, Tehran University of Medical Sciences, Tehran, Iran

**Keywords:** structured radiology report, information needs, minimum data set, the electronic health record system in Iran

## Abstract

**Introduction:**

In order to better designing of electronic health record system in Iran, integration of health information systems based on a common language must be done to interpret and exchange this information with this system is required.

**Background:**

This study provides a conceptual model of radiology reporting system using unified modeling language. The proposed model can solve the problem of integration this information system with the electronic health record system. By using this model and design its service based, easily connect to electronic health record in Iran and facilitate transfer radiology report data.

**Methods:**

This is a cross-sectional study that was conducted in 2013. The study population was 22 experts that working at the Imaging Center in Imam Khomeini Hospital in Tehran and the sample was accorded with the community. Research tool was a questionnaire that prepared by the researcher to determine the information requirements. Content validity and test-retest method was used to measure validity and reliability of questioner respectively. Data analyzed with average index, using SPSS. Also Visual Paradigm software was used to design a conceptual model.

**Result:**

Based on the requirements assessment of experts and related texts, administrative, demographic and clinical data and radiological examination results and if the anesthesia procedure performed, anesthesia data suggested as minimum data set for radiology report and based it class diagram designed. Also by identifying radiology reporting system process, use case was drawn.

**Conclusion:**

According to the application of radiology reports in electronic health record system for diagnosing and managing of clinical problem of the patient, with providing the conceptual Model for radiology reporting system; in order to systematically design it, the problem of data sharing between these systems and electronic health records system would eliminate.

## 1. Introduction

Electronic Health Record is the most important tool for delivering high quality care through the sharing of health information. International research shows that the benefits of (E-Health) electronic health record increases when it is accessible and can be used by all those involved in patient care (Sadiughi, Delgoshaei, Foozonkhah, Tofighi, & Khalesi, 2006).

This record is a source of patient data that are either directly imported or transferred from external applications (Nguyen, Bellucci, & Nguyen, 2014). So it is clear that the formation of this file is very slow and time consuming to provide data that will be generated from various resources over time. The Iran EHR system represents a set of applications that run in the proper context and the feasibility of electronic health records makes it possible. In other words, the lack of an integrated health information and possibly in different parts, has led to the emergence of this system (Seidy & Vaseghi, 2011; Sistrom, Lanier, & Mancuso, 2004).

Hence, due to the decentralization of information, the better designed EHR system requires standardized and significant data exchange with the system (Seidy & Vaseghi, 2011; unknown author, 2011).

Defining The Minimum Data Set is an important step in creating a national registration system that gives the ability to identify patients to medical institutions and meet governmental requirement, Own internal needs and medical society(Moghaddasi, 2005; Karimi, Isfahani, Farzandipour, & Esmaeili, 2011).

Radiology information system is a part of the EHR Software that designed to film storing, Interpreting and presenting radiology reports, recording and retrieval of patient data and Radiology Photography and includes the following systems: patient tracking, image tracking, examination, radiology reporting, and management system. Radiology reporting subsystem designed for registering radiology findings in the computerized clinical database (Chew & Felix, 1999; Clayton, Ostler, Gennaro, Beatty, & Frerick, 1980; Dunnick & Langlotz, 2008).

In general, the reporting assumed as a process for findings and diagnosis information extracting from images exported from any kind of modalities. According to the Radiological Society of North America these reports should include the basic defined elements, such as patient identifier, interventions description, and clinical description and photography findings. In 1922, *Hickey* made a request to standardize radiology reports. Now days, after more than 80 years since, there is still the problem of variability and quality of radiology reports. According to the royal college of radiologists in London Standardization of structure, content and setting time of this report, will increase the clinical, educational, research, quality improvement, accreditation and financial functionality of them. One way to achieve this goal is the use of structured reporting systems rather than conventional reporting free texts (Sistron, Lanier, & Mancuso, 2004; Noumeir Rita, 2006; Johnson, Chen, Zapadka, Lyders, & Littenberg, 2010). The term structured reporting represents a set of computer tools that aim at reducing variability and increasing clinical use of radiologist interpretations (Dunnick & Langlotz, 2008).

Radiology Data recording is an important means to convey the results of radiologic findings by doctors and Helps them better understand the content of the reports and so has a great impact on how care is (Brenner, 2009; Kahn, Langlotz, Burnside, Carrino, Channin, Hovsepian, & Rubin, 2009). On the other hand, given that these reports are considered as an important part of the patient’s medical record, Application of Modeling Techniques and Tools that show Radiology Reporting System Data Exchange with EHR in the graphical form, is important. Data modeling is documenting processes and events that are done during the development and design of software applications. Data modeling tools, Simplify the design of complex systems into Understandable and simple view of data flow and processes (Chew & Felix, 1999).

In the past, different software engineering techniques and tools for the analysis of health care systems were used. Recently, the concept of object-oriented analysis and design using Unified Modelling Language systems, emerged as new tools for modeling healthcare systems (Clayton, Ostler, Gennaro, Beatty, & Frederick, 1980).

Unified modeling language, is an object-oriented modeling language that used to describe the concepts of object-oriented systems (Dunnick & Langlotz, 2008). It typically includes a number of visual diagrams to describe the structural and behavioral features of the software (Brenner, 2009). The advantages of this language, are standard communication and Stable method for documentation (Dunnick & Langlotz, 2008). Since For widespread adoption of electronic health records, it is essential that with accurate design and software analysis, the standardization process is coordinated with the implementation, thus object-oriented design and analysis is a good tool for modeling complex software systems as well EHR system is (Brenner, 2009). The purpose of establishing an electronic health records system in Iran is the integration of local information systems, Due to the distribution of these softwares, analysis and exchange of information is not possible (Seidy & Vaseghi, 2011). Utilizes the Unified Modeling Language, that in fact is the standard for modeling information and processes, Systematic design of radiology reporting system can be done and by developing a conceptual model based on the Unified Modeling Language, The problem of information exchange between this system and electronic health records system be removed. In This study we assessed the information needs of users and by determining the MDS, Use case diagram for a better understanding of the operational requirements of the system were plotted.

## 2. Methods

This study was an applied and a descriptive cross - sectional study that in the first half of 2013 has been carried out. The study population includes users of Radiology reporting systems in the one of general hospitals, imaging center, consisting of 13 radiologists, three anesthesiologists, three Insurance Expert and three GP. Due to the small number of users, the sampling was not done. A questionnaire was used to collect data that made by the study of literature on the radiology report content and format standards and related articles on the laboratory, mortality, the flu and myocardial infarction data exchange with EHR system (unknown author, 2011; Shahmoradi, Torabi, & Safdari, 2012).

The questionnaire included a list of required data in the radiology reporting system in the eleven parts include: Management information, demographics, insurance, imaging procedures, anesthesia, history of previous actions, radiologist observations, radiologists interpreting, annex information signs and approval of responsible persons. For questionary validation, the content validity method was used. To assess reliability, test-retest method was used. In this way, from the same group out of study population, eight were selected and they were asked to complete the questionnaire. This work was performed twice with an interval of ten days. The correlation coefficient was determined to be 86% and the reliability of the questionnaire was confirmed. Questionnaires were used for completion by study population and they were asked to make choices based on their degree of importance from one to ten to rate So that the greatest score was 10 and a score of 1 was assigned to the least important. Finally, analysis of the data by using descriptive statistics were performed by SPSS application. According to experts, the collection of data elements for the radiology reporting system was determined and rated. The mean values were calculated for each data element and its associated descriptive tables were drawn. The following were identified as priorities:

priority 1 = average 9-10
Priority 2= average 8-8.99
Priority 3 =average 7-7.99
Priority 4 = average 6-6.99
Priority 5 = average 5-5.99
Priority 6 =average 4-4.99

To determine the minimum amount of data, data elements were selected with at least 5 and According to the relevant articles in the field of radiology minimum data reporting system and similar researches in the field of information exchange with electronic health records system, the MDS of these reports were provided. Also the use case diagrams were drawn up via Visual paradigm which is a non-commercial and open source software and is based on UML 2.0.

## 3. Results

67% of insurance experts, 33% of general practitioners, 67% of anesthetists and 54% of radiologists in the research population were men And the rest were women. Overall, more women than men were in the study population. The largest age group (55%) belonged to the age group below 40 years and most participants had experience of less than 10 years (about 55%). Table 1 shows the minimum data set for the radiology reporting system to exchange with the electronic health records.

**Table 1:** 

Data element			Data element		
	Number of encounter		Administrative data set	Name	Imaging facility
	date of service			identifier	
	time of service			address	
				first and last name	referring physician
	Place of stay			identifier	
	Informed consent			specialty	
Demographic data set	first and last name			address and phone number	
	father name			time, date and digital sign	
	medical record number			first and last name	admitting physician
	birth date			identifier	
	birth place			time, date and digital sign	
	educational level			first and last name	radiologist
	identifier			gender	
	age			time, date and digital sign	
	weight			first and last name	anesthesiologist
				identifier	
				time, date and digital sign	
	identification number			admission date	
	national code			admission time	
	geographic information(the country, province, city, town, village)			admission type(inpatient/ambulatory/emergency)	
	postal code			admission Ward	
	patient address			reason for encounter	

Data element				Data element		
Radiology data set	Encoded findings				patient phone number	
	Image annotations			Insurance data set	Serial number of the patient’s insurance	
	nature of complication	Complications (including contrast reactions)			Expiration of patient insurance	
	treatment				insurance type	
	Recommendation				insured number	
	Assessment				insurance Box	
	Plan				Insurer Name	
	radiologist’s diagnosis				Insurer ID	
	Date of previous exams reviewed				insured name	
	type of previous exams reviewed			Clinical data set	primary diagnosis	
	Pertinent observations from prior examinations				ICD-10 according to primary diagnosis	
	radiologist’s Textual description				medical	history
	Attached Image				social	
	date of image acquisition				family	
	Time of image acquisition				surgery	
					heart status	
					risk factors	
	name of imaging procedure				allergies	
	ICD-9-CM code according to procedure				pregnant	
	CPT code according to procedure				clinical query	
	procedure technique				final diagnosis	
	image identification code				ICD-10 according to final diagnosis	

Data element				Data element		
	Patient Status in the Beginning of anesthesia				imaging device	
	End of anesthesia. Time				device setting	Image acquisition parameters
	Patient Status At The End Of anesthesia				patient position	
	start of procedure time				Interventions (e.g., Valsalva maneuver)	
	End of procedure time				name	Substances administered
	B.P	vital sign			dose	
	P.R				route of administration	
	R.R				time of administration	
	T				Radiation dose	
	agent such as O2, N2o, D-Tube, etc.				anatomical site	
	Fluids				Attestation of physician presence / supervision	
	Monitoring				confirm correct patient, procedure, and site	
				Anesthesia data set	date of anesthesia	
					Pre-procedure Diagnosis	
					procedure name	
					General	anesthesia
					Local	
					anesthesia time	
					start of anesthesia time	

Based on the data analysis method described in the research method, Data elements with more than 5 priorities in the model were included in the model. Use case diagram reporting system for information exchange with electronic health records system describes that What is the reporting system should be done and by whom. In this diagram, radiologists, surgeons, anesthesiologists and Compliance Officer, as the main actor and an expert in insurance, patient insurance system, coding systems and electronic health records systems as another actor of the model was determined. Operations that were considered in this diagram include: Register, signing-in, editing reports, confirming the report, adding new information to report, deleting report, searching reports, viewing reports, share reports with external systems, reminders, digital signatures, clinical data recording, Anesthesia data recording, Reception and logout.

## 4. Discussion

Designing an information system is vital for organizations because each organization has a comprehensive, timely, and correct data that access to these data in the shortest possible time, provides the organization’s great success. Since one of the important functions of clinical information systems is to meet the information needs of professionals, in designing these systems and presenting the model of them, the assessment of end-users is necessary. This information needs should be considered to establish a standard data collection system (Boonn & Langlotz, 2009). Based on these findings, the reporting system minimum data set for the exchange with the electronic health record system were included: management, identity, insurance, clinical and imaging examination data and If the patient is put under anesthesia, System designers should also consider the anesthesia in the minimum data set. Information Standards Board (ISB), in response to the lack of accurate data for diagnostic imaging examinations, for patients in the National Health Service in England, has introduced Minimum Data Set for diagnostic imaging in the three parts: demographic, references and imaging data (unknown, 2013).

Radiology Schools.com, that is a comprehensive website to provide quality services to students and academic community, cited the main body of radiology reports in five parts: information about materials and methods, findings, clinical problems, differential diagnosis, potential limitations and comparative information. Radiology Society of North America, Australia and New Zealand are also have mentioned these elements in different terms (Plumb, Grieve, & Khan, 2009; Shahmoradi, Torabi, & Safdari, 2012; unknown author, 2011). Results showed that radiologists need clinical information when they are interpreting radiology images, because this information has an effect on how they interpreting and Contribute to patient care management and facilitate an accurate diagnosis by a physician (unknown, 2013). In all studies, importance of existing clinical information and medical history of patient was cited. Also, given that one of EHR stakeholders are insurance companies that profit from the results of the implementation of an electronic health records system by reducing costs due to reducing duplicate or unnecessary diagnostic interventions, preventing abuses caused by current insurance systems and analysis of financial data alongside clinical data. Therefore, to achieve this, insurance information must be included in the structured radiology reports (Board of the Faculty of Clinical Radiology, 2006). In general, according to studies conducted in other countries and the results of this study, most of introduced MDS are divided into two groups: clinical and nonclinical data.

Management, identification and insurance information are included in the non-clinical data and clinical information, radiology and anesthesia information are included in the clinical data. It should be noted that establish minimum data for radiology reporting systems is necessary for the exchange with the electronic health record systems because these reports are an important part of the medical record and are helpful in the diagnosis and management of future clinical problems and are a good source for research (Plumb et al., 2009; Board of the Faculty of Clinical Radiology, 2006). In the modeling stage, collaboration and comments of all users of this system in defining minimum data set, are necessary.

## 5. Conclusions

In this research, by providing use cases and standard minimum data sets, a model of radiology reporting system for the exchange with the electronic health record systems in Iran based on the user’s requirements was proposed. Since during the development and design of software applications, documentation of processes and events required for modelling data, So in order to design a better reporting system for the exchange with electronic health records system, scenario tables and accurate determination of system processes that created during a radiology report and also processes for the exchange of this report with other external systems, are necessary. Also, in this study with identifying the key data elements for reporting system, class diagram was drawn and by specifying processes in the system scenarios, use case diagram was drawn, Then the obtained use case diagram was used to draw other diagrams such as: Activity diagram, sequence diagram, collaboration diagram, state diagram. Hence it is suggested that to develop a reporting system for radiology, firstly the users’ needs should be identified and analyzed and then modelled as use case diagrams. Then, based on use case diagrams, the design team starts their work and by drawing other diagrams of reporting system provide an integrated picture of radiology reporting system so that the health system developers with understanding customer needs can create a high quality system.
